# Role of Phytochemicals in Cancer Chemoprevention: Insights

**DOI:** 10.3390/antiox10091455

**Published:** 2021-09-14

**Authors:** Blassan P. George, Rahul Chandran, Heidi Abrahamse

**Affiliations:** Laser Research Centre, Faculty of Health Sciences, University of Johannesburg, Doornfontein, Johannesburg 2028, South Africa; rahulc@uj.ac.za (R.C.); habrahamse@uj.ac.za (H.A.)

**Keywords:** chemoprevention, antioxidants, phytochemicals, carcinogenesis

## Abstract

Cancer is a condition where the body cells multiply in an uncontrollable manner. Chemoprevention of cancer is a broad term that describes the involvement of external agents to slow down or suppress cancer growth. Synthetic and natural compounds are found useful in cancer chemoprevention. The occurrence of global cancer type varies, depending on many factors such as environmental, lifestyle, genetic etc. Cancer is often preventable in developed countries with advanced treatment modalities, whereas it is a painful death sentence in developing and low-income countries due to the lack of modern therapies and awareness. One best practice to identify cancer control measures is to study the origin and risk factors associated with common types. Based on these factors and the health status of patients, stage, and severity of cancer, type of treatment is decided. Even though there are well-established therapies, cancer still stands as one of the major causes of death and a public health burden globally. Research shows that most cancers can be prevented, treated, or the incidence can be delayed. Phytochemicals from various medicinal plants were reported to reduce various risk factors associated with different types of cancer through their chemopreventive role. This review highlights the role of bioactive compounds or natural products from plants in the chemoprevention of cancer. There are many plant based dietary factors involved in the chemoprevention process. The review discusses the process of carcinogenesis and chemoprevention using plants and phytocompounds, with special reference to five major chemopreventive phytocompounds. The article also summarizes the important chemopreventive mechanisms and signaling molecules involved in the process. Since the role of antioxidants in chemoprevention is inevitable, an insight into plant-based antioxidant compounds that fight against this dreadful disease at various stages of carcinogenesis and disease progression is discussed. This will fill the research gap in search of chemopreventive natural compounds and encourage scientists in clinical trials of anticancer agents from plants.

## 1. Introduction

Cancer is a condition where cells anywhere in the body behave abnormally and multiply in an uncontrollable manner. There are over 200 different types of cancer. The symptoms and signs of cancer vary based on the size, location, stage, and cancer types. The cancer type and staging can be determined by biopsy outcomes. The treatment protocols vary based on the type and severity of the tumor. Surgery, chemotherapy, and radiation are the widely used conventional cancer treatment modalities. There are many complementary and alternative treatment modalities for cancer including herbal medicine. Chemoprevention is an approach with great potential in controlling the incidence of cancer. Chemoprevention works in different ways to stop, delay, and control cancer incidence and progression [1]. Many products from natural and synthetic agents were employed to prevent carcinogenesis or metastasis of cancer [2]. Chemoprevention research has noticeably increased with advanced insight into carcinogenesis and the detection of potent molecular targets to hinder the process of carcinogenesis. Worldwide, every year around 11 million people are diagnosed and out of which 6.7 million people die due to cancer. Breast, lung, and colorectal cancers are the most common cancers diagnosed globally and the mortality rate of lung, stomach, and liver cancers are the highest among all cancers [3]. The drawbacks of modern cancer treatments, disease severity, increased mortality rate, and loss of patient quality of life upon treatment show the need for a new and alternative herbal approach in disease control and prevention.

Chemoprevention is one of the growing areas of anticancer research with the focus on various interventions such as nutritional factors, biological and pharmacological. There are three different approaches involved in cancer chemoprevention: primary, secondary, and tertiary chemoprevention. First one involves interventions intended to prevent healthy individuals from showing high-risk features, such as genetic predisposition to prevent the development of certain cancer. Second type is mainly developed for treating premalignant tumors to prevent cancer progression (example: colon polyps, skin actinic keratosis, cervical dysplasia, lung metaplasia). Third approach is to support patients with cancer history to prevent further cancer relapse or the development of new primary tumors [3]. Nutrients, and other dietary factors are importantly linked with the risk of developing various cancer types. Manson in 2003 reported that around 35% of global cancer mortality rates are directly associated with dietary factors [4]. Recently, dietary polyphenolic compounds were reported to possess anticancer benefits and other related pharmacological activities and received a great deal of attention for their health benefits. Polyphenolic compounds are found in majority of fruits and vegetables. Based on the structural complexity, chemical nature and structure of the polyphenols, they are categorized into 10 classes with over 8000 compounds [5]. Among the pool of dietary polyphenols, flavonoids and phenolic acids are the most common and account for about 60 and 30%, while phenolic acids, flavonoids, stilbenes, and lignans are the most widely occurring plant polyphenols. This review discusses the concept of chemoprevention with reference to the plant-based chemopreventive agents, their mechanism of action, related signaling molecules, and the role of antioxidant compounds.

## 2. Cancer Chemoprevention: Rapidly Growing Field

The characteristic feature of cancer cells is their ability to grow faster than normal cells. Most of the chemotherapy agents are designed in such a way that they target these fast-growing cells and block, kill, or slow down their growth. Nevertheless, these chemotherapy drugs adversely affect or kill normal healthy cells too. Due to this reason, the patient experiences severe side effects, and the therapy efficacy will be reduced or limited. Carcinogenesis progresses through various molecular mechanisms and signalling molecules. The chemoprevention of cancer fosters the utilization of natural and artificial mediators to disrupt carcinogenesis by stopping or defeating the precise molecular signaling mechanisms [6]. These mediators are mainly classified into two: blocking and suppressing agents. Chemoprevention using medicinal plant-derived compounds is an advanced cancer research field that concentrates on prevention through nutritional mediations. Development of a chemopreventive agent from a basic biological observation to clinically efficient anticancer compounds or agent is challenging. The chemoprevention process must always be safe and lengthy, and the compounds used must be acceptable for long-term administration to healthy and elderly patients, as well as those with comorbidities.

There are several epidemiological studies that reported that consistent fruit and vegetable intake significantly reduces the possibility of the incidence of many cancers [7]. Bioactive compounds from medicinal plants or from herbal diets, dietary phytochemicals lately showed their significant role in cancer chemoprevention [6], leading to the development of a new and alternative method of cancer prevention and therapy [8]. Several phytochemicals underwent clinical trials to investigate their possible cancer chemopreventive effects [9]. Wattenberg in 1985 classified chemopreventive agents into inhibitors of carcinogen formation, blocking agents, and suppressing agents. The first type prevents the carcinogen formation from the precursors, and the second type prevents carcinogen-induced mutations by blocking specific metabolic carcinogen activation pathways, and it also enhances the detoxification process by trapping specific reactive oxygen species (ROS). Finally, the third type prevents the tumor progression by stopping cell proliferation and differentiation, and by inducing apoptosis, autophagy, necrosis, etc. Most of the bioactive compounds of plant origin block the cancer development process by any of these pathways. Chemopreventive compounds were also shown to enhance the effectiveness of conventional chemotherapeutic drugs. The important aim of cancer chemoprevention is to reduce the early-stage cancer risk and to prevent the secondary spreading of tumors [10,11].

**Figure 1:**
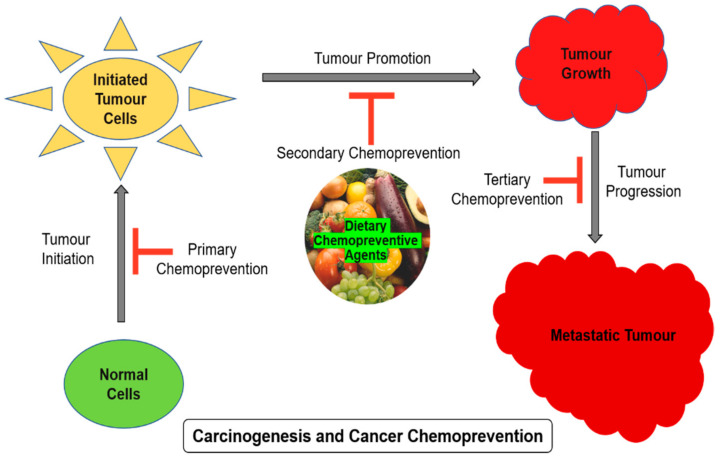
Stages of carcinogenesis and chemoprevention.

Carcinogenesis is a multistep process involving multiple genes and several genetic alterations that are expected in a normal healthy cell to develop into a tumor cell. The process of carcinogenesis, or cancer cell formation, is often divided into three steps such as initiation, promotion, and progression. The number of hereditary changes engaged in these different steps is not yet fully verified. The first step entails the initiation of an irreversibly distorted cell and is often associated with a mutation and different initiation pathways. During the second stage, the initiated mutated cells expand and form a visible mass of cells, which are probably a nonmalignant lesion. The promotion stage certainly involves epigenetic factors that affect the multiplication of the initiated cells. The detailed mechanism involved in the second stage of carcinogenesis is not well-understood. The final product of promotion is usually nonmalignant or benign cells, or sometimes preneoplastic cells. These benign cells undergo a couple of extra genetic alterations during the progression stage into neoplastic cells. The final stage in carcinogenesis, i.e., progression involves in the formation of malignant tumors from nonmalignant benign tumors, are different from other earlier two steps. Stem cells play a vital role in the initiation of carcinogenesis through various factors such as physical, chemical, or biological, including viruses. Such initiated cells will then be exposed to a promoting factor to stimulate the full neoplastic cell formation, and the sequential steps are important in the malignant transformation of preneoplastic cells [12,13]. The carcinogenesis process in a multicellular animal is the consequence of various chemical, physical, biological, or genomic changes in the cells. Though carcinogenesis is mostly driven by mutation, several other factors are also involved in this development. Chemoprevention is a pharmacological intervention approach to arrest or reverse the process of carcinogenesis. Chemoprevention influences and stops the carcinogenesis process in each stage as shown in Figure 1. 

## 3. Role of Plant Polyphenols in Chemoprevention of Cancer

Many plant-derived products are used for the treatment and management of various diseases. Several purified plant products are available in the market for use, and such products are either entire plants or parts of the plant available in powder, capsule, or liquid forms. These plant products can be consumed as food, tablets, prepared in tea, used as gels to apply over the skin, and sometimes can be used in bathing water. Even though plant products were used for centuries in anticancer and cancer chemopreventive roles, investigation of active compounds from plants drove interest in the last few decades. Nevertheless, appropriate prudence must be held during the use of such herbal products in cancer patients because some of the herbal products may interfere with the conventional treatments patients undergo, or the dosages of the plant products may cause adverse effects on the patients’ health [14,15,16,17].

One of the main drawbacks of anticancer chemotherapies is the resistance developed during the continued therapies. Recent research is focused on this area to find out the resistance genes and drug resistance processes. There are some reports on the use of herbal products in combination with conventional anticancer drugs, and the outcome showed these herbal combinations resensitize the chemotherapy resistance established after the continued use of the anticancer chemotherapeutic drugs. Therefore, these combination therapies using herbal product anticancer drug conjugates improve the healing efficacy and result in the desired treatment outcomes. The herbal combination chemotherapies also showed significantly reduced undesirable side effects of synthetic drug-based chemotherapies [18,19]. The dietetic phytochemicals are widely used in the chemoprevention process, and this idea of cancer prevention is achieving increased research focus due to the diverse chemical components in plants, complex structure of chemicals, characteristic biological effects, cost-effectiveness, easy accessibility, and reduced or less toxic side effects. Phytocompounds can alter various molecular signaling pathways and impart protective role in cancer chemoprevention. The dietary chemopreventive phytochemicals not only played an important role in cancer prevention, but they were also employed as a primary competitor as a natural lead compound in the development of potent cancer chemotherapeutic drugs. Polyphenolic compounds among the phytochemicals have a significant role in cancer therapies and prevention due to their prominent role in interfering carcinogenesis at the initiation, promotion, progression stages, etc. Phytochemicals lead to the alteration of proteins in different signal transduction pathways and integrate with distinct molecular signals to exert the definitive chemopreventive and/or chemotherapeutic role [20]. 

**Figure 2:**
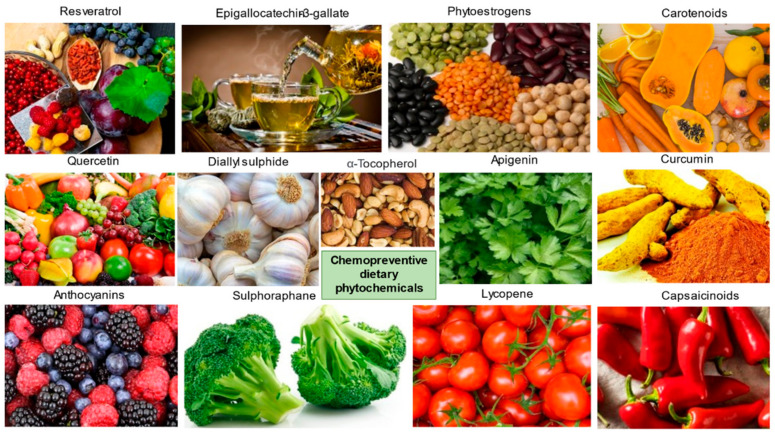
Common examples of chemopreventive dietary phytochemicals.

Environmental factors including diet are one of the major causes of the development of cancer. Nutrients, and other dietary elements are strongly correlated to the risk of developing various cancer types. Research investigations showed that one-third of cancer mortality rates are associated with diet and nutrition [4]. Phenolic acids, flavonoid compounds, stilbenes, lignans, etc., are the commonly appearing polyphenolic compounds in plants; among them, flavonoids and phenolic acids account for about 90% of dietetic polyphenols. Based on the complexity of molecular and chemical structure, there are around 4,000 different types of flavonoids reported. The dietary sources of some of the important chemopreventive polyphenolic compounds are shown in Figure 2. Various clinical studies showed the positive correlation between the intake of polyphenolic compounds or flavonoids as food or drinks rich in polyphenols and reduced cancer incidence, lowered the risk of developing different cancer types, and decreased cancer relapse [21,22,23,24,25,26,27]. Most of the potent chemopreventive polyphenols disrupt or reverse carcinogenesis through initiation, promotion, and progression steps via the network of intracellular signaling molecules [28]. Therefore, the anticancer and chemopreventive role of nutritive and dietary polyphenols are based on the combination role as cytoprotective and cytotoxic efficacies. They protect the normal healthy cells, while they kill or are toxic to the premalignant and neoplastic or malignant cells. 

Phenolic compounds of plants alter carcinogenesis through various pathways and their efficacies will vary based on the type of tissue and dosages used. Though there are some clinical studies reported on the role of polyphenols, many of the anticarcinogenesis roles of nutritive polyphenolic compounds are studied in in vitro and in vivo models. Studies showed the safe doses of green tea extract are daily up to 4.2 g or 1.0 g thrice daily intake to the patients with advanced stage of cancers, and 3 g per day for patients with advanced lung cancers. In these studies, the toxicity observed was directly linked to the caffeine (neurological and gastrointestinal effects) doses [29,30]. Another study reported the anticancer activity of green tea based on the expression of molecular biomarkers. The decreased levels of PSA and urinary 8-hydroxydeoxyguanosine markers were noted in liver and prostate cancer patients with a 6 g/m^2^ intake of green tea daily [31,32]. Some of the common medicinal plants used in the chemoprevention of cancer, their therapeutic role, and cancer types treated or prevented are listed in Table 1.

## 4. Natural Chemopreventive Agents in Clinical Setting

Many natural compounds were widely examined for their possible use in the prevention of cancers over the years. The increasing volume of in vitro and in vivo data on the cancer chemopreventive and chemotherapeutic outcomes of plant-derived compounds urged scientists to do clinical trials focusing on the pharmacokinetics, efficiency, and safety of the phytocompounds. 

### 4.1. Curcumin

Curcumin is a potential natural compound that originated from *Curcuma longa* and is widely used for cancer chemoprevention. Apoptosis induction, inhibition of molecular signals, free radical scavenging, and inhibition of inflammatory responses on tumor microenvironment are the various molecular mechanisms by which curcumin exerts chemoprevention of cancer. Curcumin exhibits ideal chemopreventive features, such as low toxic effects, cost-effectiveness, and easy accessibility. In different continents, especially in Asia, the powder form of rhizome of the *Curcuma* plant was used widely as a spice and as a coloring and flavoring agent in many foods, and it also showed anti-inflammatory effects. Diferuloylmethane (1,7-bis- (4- hydroxy-3-methoxyphenyl)-1,6-heptadine-3,5-dione) or curcumin is the chief pigment of turmeric, with strong antioxidant and anti-inflammatory activities [60]. Turmeric contains many bioactive compounds of which curcuminoids such as curcumin, tetrahydrocurcumin, bisdemethoxycurcumin, and demethoxycurcumin are common, and the levels vary based on the species type, farming, and rhizome treating conditions. Tetrahydrocurcumin is the key naturally occurring curcuminoids compound, and it is of great interest in anticancer research due to its increased solubility in water. They are chemically stable and showed maximum bioavailability with strong antioxidant activity. Anticancer activities of tetrahydrocurcumin are linked to many molecular pathways such as they modulate oxidative stress created in cells, reduce inflammation and proliferation, and induce immunity and cell death [61].

The major molecular targets of curcumin that help in cancer cell death are cyclooxygenase-2 (COX-2), nuclear factor kappa B (NF-kB), tumor necrosis factor-alpha (TNF-a), and cyclin D1. The molecular targets exert substantial anti-inflammatory and anti-tumorigenic properties in different clinical and preclinical studies [62,63]. Many in vitro studies also showed different mechanisms of curcumin in the antiproliferation of cancer cells. Cyclin D1 and CDK-4 expressions were significantly reduced in breast and skin cancers after curcumin treatments [64]. Curcumin also downregulates the expression of angiogenic genes including VEGF, angiopoietin and MMP-9, and MMP-3; this effect was mediated by AP-1 inhibitory action [65,66] a promising in cancer therapeutic and chemopreventive approach. The chemopreventive action of curcumin is altogether mediated by several biochemical and molecular pathways that regulate apoptotic cell death and the role of different types of transcription factors and enzymes.

### 4.2. Resveratrol

Grapes, berries, and many other plant products exhibit strong polyphenolic compounds are rich in resveratrol (3, 5, 4′-trihydroxy-trans-stilbene). Not only in cancer research but many other clinical research data also showed that resveratrol has significant effects with many age-related diseases, such as diabetes, neurodegenerative disorders, arthritis, and coronary and pulmonary illnesses. Reports suggest that resveratrol modifies all the different steps in carcinogenesis, such as initiation, promotion, and progression. They alter various signaling mechanisms to reduce the cancer cell multiplication and progression, inducing programmed cell death and reducing inflammatory responses and angiogenesis, and also stopping the metastatic spread of tumors. They modulate multiple signaling pathways of the cell cycle, inflammation, and apoptosis [67,68,69,70]. One of the promising roles of resveratrol is how it significantly decreases the toxic side effects associated with therapies and enhances the cancer therapeutic effects. It can also be employed in the treatment of some autoimmune diseases [71,72,73,74].

Side effects of chemotherapies were always a serious complication in the application of chemotherapy drugs in clinical practice. Resveratrol is a safe and potent natural compound that showed a multifunctional role which has a protective role against chemotherapy side effects with strong chemopreventive and chemotherapy properties [75,76]. Resveratrol significantly reduced the accumulation of arsenic and decrease the arsenic-induced toxicities in renal cells. They also inhibit the arsenic trioxide-induced oxidative stress, and decline the arsenic accumulation in hepatic cells and increase antioxidant enzyme effects to prevent arsenic-induced toxicity in liver [77]. The promising antioxidant role of resveratrol also helps to protect acetaminophen-induced liver toxicity, as well as cisplatin-induced gastrointestinal tract disorders by improving the tubular dilation and cell vacuolization of kidney tubes [78,79]. The topical application of resveratrol inhibits UVB-mediated skin edema and reduces hydrogen peroxide production and leukocyte infiltration in mice. This study led found that the long-term application of resveratrol before and after the treatment significantly reduced the tumor incidence or delayed the start of oncogenesis, while the short-term application led to the inhibition of cellular proliferation [80,81]. Some studies reported that regulatory cell cycle signaling molecules and apoptotic inhibitory proteins expression after resveratrol treatments showed inhibition of photocarcinogenesis [82,83].

### 4.3. Apigenin 

A flavonoid rich in fruits and vegetables with chemopreventive action is apigenin (4′, 5, 7, -trihydroxyflavone). Antiangiogenic properties of apigenin are linked to the regulation of signaling pathways, induction of apoptosis, prevention of cancer cell transformation, and cell cycle arrest [84,85,86,87]. Apigenin has an inhibitory effect on tobacco smoke-related cancers and HPV infections. They substantially reduced HPV-altered human prostate cells by cell cycle alteration and apoptotic induction [88], and this effect was due to the inhibiting action of apigenin on NNK, a tobacco-specific carcinogen through focal adhesion kinase (FAK) and extracellular regulated protein kinase (ERK) signals [89]. Furthermore, they specifically inhibit HPV-positive HeLa and SiHa cells while reduced toxic effects are observed in HPV-negative C33A and HaCaT cells [90].

There are many in vivo studies that reported the chemopreventive role of apigenin. The dosages, different modes of administration, and frequencies of treatments, etc., were studied on various animal models. The in vivo results showed the inhibition of phosphoinositide 3-kinase (PI3K)/Akt/Forkhead box O-signaling pathway [91], and apigenin reduced Her2/neu protein expression in breast cancer mice models [92]. In a xenograft tumor model, the intake of apigenin also reduced the serum-immunoglobulin F-I concentration, and stimulated apoptotic cell death and cell cycle arrest at different phases [93]. The interruption of apigenin in the NF-kB pathway is responsible for prostate cancer inhibition [94]. A clinical study was conducted to investigate the absorption of apigenin systemically after the intake of parsley rich diet, the results of which showed improved biological antioxidant levels, such as SOD and GR, whereas the GPx, CAT activities seemed to be reduced in blood cells [95]. Apigenin also showed other bioactivities, such as reduction in plasma level LDLs, platelet aggregation, and cell proliferation [28,95,96].

### 4.4. Epigallocatechin Gallate

Green tea is rich in a strong antioxidant chemopreventive compound epigallocatechin gallate (EGCG). The chemical structure of EGCG consists of three attached heterocyclic rings, and electron delocalization led to the free radical scavenging [97]. Tea catechins including EGCG have redox properties and react with reactive oxygen species. The metal chelating abilities of EGCG helps in the prevention of ROS production and catechin oxidation process [98,99]. The chemical structure of EGCG imparts antioxidant properties and the air oxidation process with ions and leads to the generation of unstable dimers of catechin. Though EGCG has many health benefits, it showed low bioavailability due to increased instability, low distribution in the digestive tract, and active efflux properties [100,101,102,103]. Due to these drawbacks, the effect and application of EGCG in human trials is reduced. Recently, many clinical trials were conducted on controlled pharmacokinetic factors to choose highly suitable dosages and administration modes for proper EGCG intervention [103].

Several in vitro studies conducted to investigate the different mechanisms of EGCG chemoprevention. EGCG shown prominent regulatory effects on various signalling pathways such as JAK/STAT, MAPK, PI3K/AKT, Wnt, Notch, NF-κB and AP-1 [104,105,106,107,108,109,110,111]. The bioactive compounds of green tea also established tumour suppressor activities, including p53, p21, p16 and Rb [112,113,114,115]; these activities play a vital role in the prevention of cancer [116,117]. Another important property of EGCG is its regulatory role on several receptors in host; for example, EGCG control 67-KDa laminin receptor activity, formerly recognized as extracellular matrix as part of the translational assembly and as surface receptors [118,119]. Furthermore, they control the androgen receptor activity in prostate tumors [120] and the estrogen receptor action in the mammary tumors [121].

### 4.5. Genistein 

Legumes produce an important isoflavone known as genistein (4′, 5, 7-Trihydroxyisoflavone), commonly called phytoestrogens with a similar structure to human estrogen. They are ketone or nonketone polyhydroxy polyphenolic compounds of leguminous plants [122]. Genistein exhibit a wide range of biological properties including cancer chemoprevention. Epidemiological studies showed that in Asian countries, the incidence of certain types of cancers such as breast and prostate cancers is lower compared to that of western countries due to diets rich in soy and soy products [123]. The report says the genistein was first isolated from *Genista tinctoria*, Fabaceae family, in 1899, but the most abundant amounts of genistein are sourced from legumes [124].

Biochemical properties of genistein mediate many health benefits including antitumor. Genistein phytoestrogen competes with 17β-estradiol in estrogen binding tests in vitro cell cultures. The report says genistein blocks the proliferation and multiplication of estrogen and androgen receptor-positive and negative mammary and prostate tumor cells in vitro [125]. Genistein blocks PTK signaling mechanism via protein-tyrosine kinase (PTK) inhibition, which indirectly suppresses the proliferation of cancer cells [126]. Other important molecular cancer targets of genistein are topoisomerase I and II [127], 5α-reductase [128], and protein histidine kinase [129]. Inhibition of these targets also imparts antiproliferative and apoptotic induction of cancer cells. Genistein showed strong antioxidant potential and protects the cells from ROS action via free radical scavenging, and it also inhibits the stress response-related genes expression and reduces their role in carcinogenesis [130]. Genistein is a strong inhibitor of cell survival pathways, such as NF-κB and Akt [131], and these inhibitory effects play a major role in the apoptosis induction in genistein-treated cells.

Some of the most well-known plant-derived chemopreventive agents, their chemical structure and chemopreventive mechanisms are listed in Table 2.

## 5. Phytochemicals Induced Chemopreventive Mechanisms

### 5.1. Antiangiogenesis and Metastasis

The physiological process by which new blood vessels are formed from the old ones is called angiogenesis. This process is a vital step in the growth, invasion, and spread of tumors. There are many phytochemicals including phenolics that act as angiogenesis inhibitors to block tumor cell growth and multiplication. The preliminary mechanisms of antiangiogenic properties of phenolic compounds are well understood in many in vitro and in vivo studies. The antiangiogenic properties of ellagic acid, EGCG, genistein, and anthocyanin-rich berry extracts are via suppression of vascular endothelial growth factor, vascular endothelial growth factor receptor-2, platelet derived growth factor, platelet-derived growth factor receptor, hypoxia-inducible factor 1a, and matrix metalloproteases. The blocking of epidermal growth factor receptor, vascular endothelial growth factor receptor, and platelet-derived growth factor receptor phosphorylation also impart in antiangiogenesis [136,137,138,139].

Many phenolics show a variance of effects on antiangiogenic factors of tumor and healthy normal cells. Green tea phenolic EGCG can chelate ferrous ions and prevent hypoxia-inducible factor-1a-induced cancer cell growth. In vitro studies showed a decrease in hypoxia-inducible factor-1-mediated transcription and hypoxia-inducible factor-1a protein level under normal oxygen levels after treatment with EGCG in prostate tumor cells [140]. Kaempferol, quercetin, myricetin, and galanin suppress the vascular endothelial growth factor-mediated human umbilical vein endothelial cells (HUVEC) tubular formation and block the U937 cell adhesion to HUVECs to prevent the angiogenesis process [141]. Flavonoids, such as naringin, rutin, apigenin, genistein, and kaempferol, decreased the vascular endothelial growth factor from human mammary cancer cells [142]. Metastasis is the process of spreading cancer cells to distal organs through lymph nodes. This process interchange with the degradation of extracellular matrix, proteolysis, cell adhesion, cell migration, angiogenesis, and invasion [143]. Many dietary polyphenolic compounds possess anti-invasive and antimetastatic properties by interfering with the tumor cell adhesion and migration via various mechanisms. However, their exact molecular mechanism and signal transduction pathways are yet to be discovered [4].

### 5.2. Apoptosis and Cell Cycle Arrest

Apoptosis is the most common programmed cell death and acts as the therapeutic target for many cancers. Several dietary chemopreventive compounds (resveratrol, quercetin, EGCG, curcumin, apigenin, chrysin, silymarin, and ellagic acid) showed the inhibition of carcinogenesis via apoptosis induction [4,28]. Cancer cells are more sensitive to these compounds than healthy normal cells [144,145,146]. EGCG mediated apoptosis induction in sarcoma cells was by the cell cycle arrest at G2/M phase, suppression of Bcl-2 and myc, expression of p53 and Bax, whereas the expression of other important targets of apoptosis such as p21, p27, Bcl-xL, mdm2, and cyclin D1 remains unchanged in sarcoma cells [147]. However, the overexpression of p21, p53 and Bax were observed along with the activation of caspases-3 and -9 and PARP cleavage to achieve apoptosis in prostate cancer cells. EGCG primarily activates and promotes the cell cycle arrest and apoptosis through p53-mediated signal transduction along with the effect of p21 and Bax [148]. Another study showed that a black tea phenolic called theaflavin stimulated the fragmentation of DNA, caspases-3 and -8, Bax expression, and Bcl-2 downregulation to achieve apoptosis [149]. Remarkably, theaflavin also stimulated apoptosis by p53 expression, altering the Bax/Bcl-2 ratio, increased release of cytochrome c from mitochondria, and activates the caspases-9 and -3 expressions in prostate cancer cells [150]. An important class of flavonoid compound, anthocyanins, showed an ability to decrease cell proliferation in colon cancer cells in a dose-dependent manner. Anthocyanins-mediated apoptotic induction in colon cancer cells was due to the DNA fragmentation and imbalance of Bax and Bcl-2 expressions. However, some bioflavonoid compounds (rutin, epicatechin, chlorogenic acid, or p-hydroxybenzoic acid) showed no growth inhibitory effect in cells [145]. Delphinidin is an anthocyanidin plant pigment that inhibited vascular endothelial growth factor-mediated cell movement and proliferation through the cell cycle arrest at G0/G1 phase. During this process, the expression of p21 and p27 increased whereas the levels of cyclin D1, cyclin A significantly reduced [151]. Additionally, the delphinidin-mediated antiproliferative effect was also caused by the early activation of extracellular signal-regulated protein kinase1/2, upregulation of caveolin- 1, and downregulation of Ras [152].

## 6. Signaling Molecules Involved in Cancer Chemoprevention

There are several factors leading to the progression of cancer, including complex interactions of signaling molecules [153]. With advancement in science and research technologies, these pathways are being unveiled to track the conditions leading to cancer progression. Molecular oncology has identified key mechanism of actions with a precise understanding of gene expression, suppression, mutations, etc., [154,155]. Several medicine and drugs performed wonders in cancer therapy. However, cancer incidence and progression can be prevented by modifying lifestyle and dietary changes [28]. These dietary components as chemopreventive agents attained much interest in resolving many medical conditions.

### 6.1. Phytochemicals Modulating Signaling Molecules

Naturally occurring chemopreventive agents, especially from plants, inspired the scientific community to conduct many experiments to investigate the underlying mechanisms of action against cancer prevention [156]. Plant-based compounds or phytochemicals interfere with cellular mechanisms including action of antioxidant enzymes, reducing oxidative stress, cell cycle arrest, apoptosis, necrosis, autophagy, and inhibit expression of genes suppressing cancer progression and lead to activation of oncogenes, modulating signaling pathways and inhibiting angiogenesis and metastasis. All these and many other pathways have targeted molecules that activate chemoprevention of cancer [157,158].

These target molecules include kinases, receptors, caspases, tumor-suppressor proteins, transcriptional factors, miRNAs, and cyclins. Due to internal or external factors, oncogenes get activated inducing carcinogenesis. However, cells initiate their self-defense mechanisms through apoptosis, cell cycle arrest, autophagy etc., by altering cell signaling pathways. Phytochemicals can accelerate these defense mechanisms on cellular matrix, cytoplasm and nucleus through multiple molecules controlling these pathways which decide cell fate [71,159,160].

### 6.2. Major Signaling Pathways

MAPK Pathways. Phytochemicals can target the extracellular signal regulated kinase (ERK) and mitogen-activated protein kinase (MAPK) pathway, which essentially regulates cellular growth and survival. These natural compounds from plants potentially control cancer progression through various mechanisms [160,161]. The major plant compounds that were reported to induce apoptosis through MAPK and ERK pathways include ursolic acid, kaempferol, resveratrol, gingerol, sulforaphane, genistein, and isothiocyanates [161,162,163].

Akt Signaling Pathways. In cancer control and progression, Akt/PI3 signaling pathway plays a pivotal role. Levels of epidermal growth factor (EGF) regulates series of molecular mechanisms including activation of NF-κB and phosphorylation of Akt leading to resistance to apoptosis and uncontrolled cell proliferation, while downstream, it leads to the regulation of caspases, Bcl-2, glycogen synthase kinase 3-beta (GSK3β), and mammalian target of rapamycin (mTOR) [164]. Alkaloids and phenolics significantly contributed to controlling the expression of these factors. Resveratrol, luteolin, luteolin, apigenin, flavone, sulforaphane, and curcumin were reported to induce anticancer activities through cell cycle arrest and apoptosis, inhibit Akt/PI3K signaling, proapoptosis, activation of FOXO3a (forkhead box O3), antiproliferation, and anti-invasion [165,166,167,168,169,170,171].

JAK/STAT Signaling Pathways. Activation of Janus kinases (JAKs) subsequently phosphorylates signal transducer and activator of transcriptions (STATs), and translocates to the nucleus, where it controls the transcription of p53, Bcl-2, cyclin D, and interlukin-6 (IL-6) involved in cell death, proliferation, and apoptosis [172]. Compounds isolated from plants significantly induce cell death in various cancer forms by inhibiting activity of JAK/STAT signaling and activating apoptotic cascades [173].

Wnt/β-Catenin Signaling Pathways. Common cancers, including that of breast, lung, colon, blood, ovary, skin, and brain, are frequently associated with abnormal signaling of Wnt/β-catenin pathway [174]. Activation of this pathway is initiated with the binding of Wnt-protein to frizzled family transmembrane receptors and accumulation of β-catenin in the nucleus leading to the activation of transcriptional factors regulating cell proliferation, survival, and migration [175,176]. Phytochemicals, such as curcumin, resveratrol and epigallocatechin-3-gallate (EGCG), inhibit the translocation and accumulation of β-catenin in the nucleus by activation of glycogen synthase kinase 3 (GSK3) [174,177,178].

Tumor Suppressor p53. Similar to other signaling pathways, p53 accounts for anticancer activities in cell. This tumor suppressor protein facilitates activation of apoptotic cascades [179]. Several phytochemicals, including resveratrol, Quercetin, EGCG, and piceatannol, were reported to upregulate p53, downregulate Akt, and induce cellular apoptosis and elevated cell cycle rest [179,180,181]. Another significant contribution is with p53, MAPK, and JNK pathway crosslink [182].

**Figure 3:**
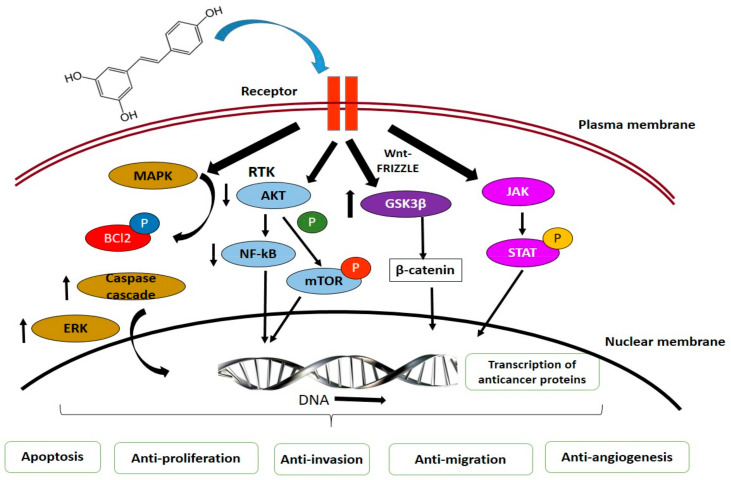
Interaction of phytochemicals with signaling molecules in cancer chemoprevention. Figure represents action of phytochemicals on MAPK, Akt, Wnt, and JAK/STAT pathways inducing cancer cell death through activation of several internal signaling molecules.

Curcumin and ursolic acid reportedly promote autophagy, wherein the cells shut down irregular cell proliferation and progression. In cancer cells, autophagy is induced through downregulation Akt/mTOR pathways [183]. Dietary phytochemicals also regulates of antitumor functions in tissues by regulating inflammation, angiogenesis, invasion, and metastasis [184]. Phytochemicals, hence through these signaling pathways, suppress cancer development (Figure 3).

## 7. Role of Antioxidants in Chemoprevention of Cancer

Many factors promote cancer development, and one among them are free radicals. These free radicals fundamentally belong to reactive nitrogen species (RNS) and reactive oxygen species (ROS). Singlet oxygen, superoxide radicals, hydrogen peroxide, and nitric oxide (NO) are known to impart potential impact on cellular mechanisms [185]. These free radicals cause damage to lipid bilayer disrupting the cellular membrane, also tampering with amino acids and DNA, thereby activating a series of enzymatic and nonenzymatic reactions inside the cell, leading to irregular gene expressions [186]. In cells, some of the ROS produced are neutralized by endogenous antioxidants in the body. However, imbalance between pro and antioxidants results in accumulation of free radicals promoting malignancy and metastasis.

The oxidative damage of DNA induces gene mutations and chromosomal aberrations, leading to failure in cell cycle arrest in G1 and diminishing DNA repair capacity. This will promote replication errors, inactivation of tumor suppressor gene, activation of oncogenes, and malignancy. The depletion of normal cell population is more evident with free radical induced resistance in cells due to incorrect DNA replication and chromosomal rearrangements leading to carcinogenesis. Studies reported activation of C-Raf-1 and K-RAS oncogenes by hydroxyl radicals. This activation initiates through N-terminal deletion of genes and GC base pair mutations [187].

To counteract these free radicals and promote cell death in tumor tissues, antioxidants are being studied widely as they neutralize free radicals that enhance cancer progression. Endogenous and exogenous antioxidants are the major forms of classified based on their source. This includes enzymes, phenolics, carotenoids, minerals, and vitamins [188]. Despite the availability of synthetic antioxidants, antioxidants from natural sources are abundant with immense health benefits. Dietary antioxidants are among the most explored ones as they can be included in daily diet which can mitigate free radical induced oxidative stress and disease [189]. Plant-derived antioxidants showed explicit potential in preventing diseases, and hence, researchers’ interest towards identifying novel antioxidants from plants are increasing.

Cancer and its subtypes are a challenging concern for most of the medical experts and researchers all over the world. However, plant-derived compounds, especially those with antioxidant properties to counteract free radicals and oxidative stress, are promising solutions in cancer chemoprevention. Moreover, these phytochemicals are proved to possess none-to-minimal toxicity towards normal cells, making them preferred agents in chemoprevention.

## 8. Possible Actions of Antioxidant Phytochemicals

Several phytochemicals induce antimigratory, anti-invasive, and antiproliferative effects on cancer cells. Free radicals induced carcinogenesis and activation of nuclear factor erythroid 2-related factor 2 (Nrf2), resulting in the accumulation of ROS and increased oxidative stress. These results in translocation of Nrf2 into nucleus once dissociated from Kelch-like ECH-associated protein 1 (KEAP1). Meanwhile, KEAP1 undergoes proteasomal degradation. In the nucleus, Nrf2 binds with Musculoaponeurotic Fibrosarcoma (MAF), initiating transcription of genes activating superoxide dismutase (SOD), glutathione-S transferases (GSTs), quinone oxidoreductase 1 (NQO1), heme oxygenase-1 (HMOX-1), suppressing free radical-induced stress, as well as DNA and protein damage, and downregulating several other signaling pathways responsible for carcinogenesis [190]. In the following paragraphs, we discuss a few important antioxidant phytochemicals with proven chemopreventive roles against cancer.

Curcumin, one of the major alkaloids found in plants of cucurbitaceae family, is known to reduce ROS-induced tumorigenesis and simultaneously protect normal tissues from ROS-mediated DNA damage [191]. Curcumin showed the ability to modulate several signaling pathways by reducing free radical load in cancer cells. Extracellular signal regulated protein kinase (ERK1/2) is one such signaling molecule involved in reverting ROS production, tumor invasion, and migration [192]. Nuclear factor kappa B (NFkB) is also activated in cancer cells in response to ERK1/2 pathway [193]. Endogenous enzymes such as catalase (CAT), superoxide dismutase (SOD), and heme oxygenase-1 are important in fighting free radical imbalance in the cells. Phenolic compounds were potentially proven to be an agent in activating these enzymes inhibiting the production of ROS in several organs and reducing metastases by altering levels of matrix metalloproteinase (MMPs), Vascular endothelial growth factor (VEGF), and Protein kinase (PKC) levels [194]. Garattini et al. studied the effective role of all trans retinoic acids and other retinoids in the prevention of breast cancer. This was attained by modulating several growth factor pathways such as epidermal growth factors including (EGF), insulin growth factor (IGF), mitogen activated protein kinase (MAPKs) and Akt signaling pathways [195]. Angiogenesis, being one of the major promoters of cancer proliferation, became a target of cancer prevention. Retinoids prevent this process of angiogenesis in cancer populations [196]. These and other classes of phytochemicals are known to eliminate cancer stem cells responsible for cancer relapse [197], inducing apoptosis and modulating gene and immune functions [198]. The preventive role of tocopherols were studied in many cell lines inducing antiproliferative, apoptotic, cell cycle arrest, and proapoptotic activity in pancreas, colorectum, lung, breast, prostate, liver, bladder, stomach, and glioblastoma [199,200,201]. Shin-Kang et al., [202] studied the mechanism behind these properties and concluded that tocols act on several signaling cascades, including inhibition of ERK, Akt, cyclins, NF-κB, P13K/Akt signaling, TGF-β, and EGF-2, upregulation of caspases 8, 6, and 9, and suppression of cell proliferation [199].

One of the major classes of compounds found in plants with known wide range of properties are phenolic compounds (PCs). They were shown to kill cancer cell lines in many ways. Similar to phytochemical actions discussed above, phenolic compounds also exert their preventive role though cell cycle arrest, apoptosis, and most importantly, scavenging free radicals responsible for carcinogenesis and its progression [203,204,205]. Phytochemicals involved in cancer chemoprevention were always a topic of controversy due to their pro-oxidant action. However, compounds from plants are sought after to effectively prevent cancer. The role of such natural compounds are shown in Table 3.

## 9. Role of Natural Products in CYP450 Inhibition

Several natural compounds or phytochemicals were proven to exert inhibitory activity towards different isoforms of P450 protein family as potential anticancer and chemopreventive therapeutic agents. Phytochemicals, such as polycyclic aromatic hydrocarbons (PAH), naphthoquinones and anthraquinones, stilbenoids, flavonoids, coumarins, alkaloids etc., inhibit the P450 enzyme activity. Mechanistically, these phytochemicals are classified as reversible (competitive or noncompetitive), quasi-irreversible, and irreversible inhibitors of P450 enzyme. Phytochemicals, such as polycyclic aromatic hydrocarbons, flavones, and coumarins, exhibit very high P450 inhibitory activity. Nonsubstituted PAHs, 5-hydroxy-2-methyl-NQ, 2-methyl-NQ, 2-hydroxy-NQ, and 1,4-NQ, 2,4,3′,5′-Tetramethoxystilbene, protoberberine alkaloids, cannabinoids etc., inhibit P450 enzymes in a competitive manner. The well-known anti-inflammatory and anticancer agent berberine acts as a noncompetitive inhibitor of P450 CYP1 family enzymes. Linderane and thujopsene terpenoids show an irreversible inhibition of CYP2 enzymes, while methylenedioxyphenyl lignans act as an irreversible inhibitor of CYP34A [214,215,216]. Resveratrol exhibits various P450 inhibitory mechanisms such as it inhibits human P450 CYP1A1 activity in a mixed-type inhibition (competitive–noncompetitive), while also acting as a noncompetitive inhibitor against P450 CYP1B1 and a mechanism-based inhibitor of cytochrome P450 CYP3A4 [214]. A unique inhibition type known as mechanism-based inactivation (MBI) is also observed in certain natural compounds. In MBI the original substrate of the enzyme undergoes additional metabolic conversion, which chemically activates the substrate to irreversibly binds and inactivates the enzyme. The natural mechanism-based inhibitors of P450 enzymes include anthraquinone derivatives (4-amino-1-chloro-3-methylanthraquinone), stilbenoids (rhapontigenin), flavones (7-hydroxyflavone, 3-flavon propargyl ether), coumarins (coriandrin), alkaloids (furafylline), etc. However, application of MBIs as anticancer agents is not recommended, as irreversible and permanent inhibition of P450 enzyme causes disturbed pharmacokinetics and pharmacodynamics of drugs metabolized by P450. This causes a significant increase in drug concentration in blood circulation, leading to toxicity [214,215]. Other than direct inhibition, resveratrol also acts as a competitive antagonist of arylhydrocarbon receptor (AhR) ligands such as dioxin by blocking the activation of dioxin-inducible genes and inhibits cytochrome P450 1A1 expression [217,218]. In an in vivo study, resveratrol was shown to abrogate the benzo[a]pyrene-induced P450 1A1 expression and its subsequent effects on B[a]P-DNA adducts formation and apoptosis induction [219].

## 10. Conclusions

Chemoprevention of cancer using plant-based compounds became a preferred approach in cancer management. Exploration of new chemopreventive phytocompounds became a central goal of anticancer research, and it also helps with finding new therapeutic targets. Specific dietary compounds explained in this review may constitute potent cancer chemopreventive agents, and the consumption of food that involves such bioactive compounds was shown to have protective and therapeutic effects on various types of cancers. The polyphenolic compounds from plants have an immunomodulatory role that identifies and destroys cancer cells by antiangiogenic effects. Chemopreventive drugs enhance chemo and radiotherapy efficacies via multiple signal transduction pathways. Since oxidative stress plays a vital role in the pathogenesis of many cancers, the antioxidant effect of dietary phenolic compounds might act as a promising strategy to prevent cancer. Dietary antioxidant phytochemicals are abundant in plants representing different classes of compounds, which exert various mechanisms of actions on tumors. Hence, chemoprevention through diets rich in plant-based antioxidants show great potential in reducing the risk factors associated with cancer progression.

## Figures and Tables

**Table 1:** Medicinal plants used in chemoprevention of cancer and their therapeutic role.

Common Name	Botanical Name	Therapeutic Role	Type of Cancer	References
Indian Gooseberry	*Phyllanthus emblica*	Immunomodulatory, cytoprotective	Breast cancer	[33]
Garlic	*Allium sativum*	Anticancer, immuno-stimulant	Oesophageal, colorectal cancer	[34,35]
Turmeric	*Curcuma longa*	Anti-inflammatory, anticancer; chemo-resistance	Breast cancer	[36,37,38]
Common mayapple	*Podophyllum peltatum*	Anticancer	Testicular, lung cancer	[39]
Heartleaf moonseed plant	*Tinospora cordifolia*	Immunomodulatory, antioxidant, anticancer	Cervical cancer	[40]
King of bitters	*Andrographis paniculata*	Immune stimulator	Leukaemia, Colon, breast cancer	[41]
Atemoya	*Annona atemoya*	Anticancer	Lung, Colon, breast cancer	[42]
Stone breaker	*Phyllanthus amarus*	Cell cycle arrest, DNA repair, anti-angiogenic	Lung	[43]
Amruta	*Mappia foetida*	Antineoplastic	Leukaemia, lymphoma, cervical	[44]
Winter cherry	*Withania somnifera*	Anti-inflammatory, antitumor, antioxidant, immuno-modulatory	Leukaemia	[45]
Himalayan cedar	*Cedrus deodara*	Apoptosis induction	Leukaemia	[46]
Heart leaved moonseed	*Tinospora cordifolia*	Cytotoxic	Cervical	[47]
Soursop	*Annona muricata*	Cytotoxic	Breast	[48]
Chestnut rose	*Rosa roxburghii*	Immunomodulatory, antiaging	Oesophageal, gastric, pulmonary	[49]
Jewel Vine	*Derris scandens*	Radiosensitizer	Colon	[50]
Penawar Hitam	*Goniothalamus macrophyllus*	Apoptosis induction	Cervical	[51]
Dong quai	*Angelica sinensis*	Cytotoxic	Leukaemia	[52]
Cang Zhu	*Atractylis lancea*	Apoptotic, cell cycle arrest	Liver	[53]
Mongolian milkvetch	*Astragalus membranaceus*	Immunomodulatory	Myeloid Leukaemia	[54]
Tea plant	*Camellia sinensis*	Antioxidant, antitumor, antibacterial, antimutagenic	Breast, lung, colon, skin	[55]
Fire-flame bush	*Woodfordia fruticosa*	Cytotoxic	Lung, colon, Liver, Neuroblastoma	[56]
Red spiderling	*Boerhaavia diffusa*	Cytotoxic, anticarcinogenic	Cervical	[57]
Umbrella cheese tree	*Glochidion zeylanicum*	Cytotoxic	Prostate, liver, colon	[56]
Black nightshade	*Solanum nigrum*	Antimicrobial, antioxidant, cytotoxic, antiulcerogenic, hepatoprotective	Cervical	[58]
Chaga	*Inonotus obliquus*	Anticancer	Lung, breast, cervical, stomach	[59]

**Table 2:** Chemopreventive agents and mechanisms.

Chemopreventive Agents	Chemical Structure	Chemopreventive Mechanism	References
Curcumin	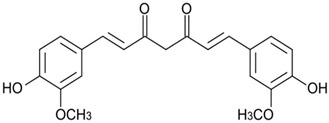	Inhibits lipid peroxidation, free radical generation, lipo- and cyclooxygenase, protein kinase C	[132]
Resveratrol	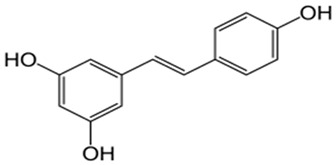	Alter signalling mechanism of cell division and proliferation, cell death, inflammation, angiogenesis, metastasis	[70]
Apigenin	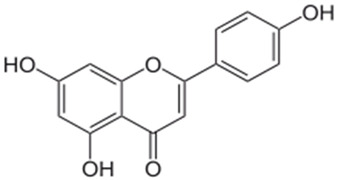	Inhibit angiogenesis, malignant transformation, cell invasion, metastasis, induce apoptosis, regulate cell cycle	[133]
Epigallocatechin gallate	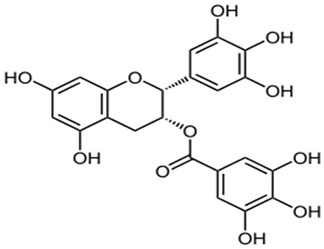	Regulate signalling pathways- PI3K/AKT, JAK/STAT, Notch, Wnt, AP-1, NFκB. tumor suppressor activities- p53; p21, p16 and Rb	[134]
Genistein	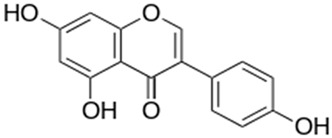	Inhibit cell growth, protein-tyrosine kinase, NF-κB, Akt signalling, PTK, regulate cell cycle regulation, induce apoptosis	[135]

**Table 3:** Gene regulatory mechanisms of phytochemicals.

Phytochemical	Action	Gene Regulation	References
Curcumin	Antiproliferation, antimigration, anti-invasion, cytotoxicity Reduces tumor invasiveness	↓ ERK1/2 and NFkB STAT	[206,207]
Epigallocatechin-3-gallate	Antiproliferation, antioxidant defence capacity Reduces exogenous oxidative stress	↓ NFkB ↑ GSH ↑ Nrf2, UGT1A, UGT1A8, and UGT1A10	[208,209]
Resveratrol	Inhibits ROS-induced proliferation and Migration Protects against (4-OHE2)- induced migration and transformation	↓ pERK, pAKT, and pNFkB ERK, NFkB and p38, MAPK/NFkB signaling	[210]
Hesperedin	Reducing oxidative stress	↓ NFkB and COX-2	[167]
Quercetin	Accelerates endogenous antioxidant enzymes, Apoptosis	GST and GPx	[211,212]
Gingerol	Apoptosis	↑ MAPK	[213]
Ursolic acid	Antiproliferation, proapoptosis, proautophagy	↓ Akt, ↓ MAPK	[171]
Apigenin, flavone, eupatilin	Antiproliferation, proapoptosis	↓ Akt	[180]

‘↓’ indicate down-regulation and ‘↑’ indicate up-regulation of genes.
